# Gender stereotypes about intellectual ability in Japanese children

**DOI:** 10.1038/s41598-022-20815-2

**Published:** 2022-10-11

**Authors:** Mako Okanda, Xianwei Meng, Yasuhiro Kanakogi, Moe Uragami, Hiroki Yamamoto, Yusuke Moriguchi

**Affiliations:** 1 Department of Psychology, Otemon Gakuin University, Ibaraki, Japan; 2 Graduate School of Human Sciences, Osaka University, Suita, Japan; 3 Department of Human Environment Design, Sugiyama Jogakuen University, Nissin, Japan; 4 Graduate School of Letters, Kyoto University, Yoshidahonmachi, Kyoto, Japan

**Keywords:** Psychology, Human behaviour

## Abstract

Japan has a large gender gap; thus, this study examined whether Japanese 4- to 7-year-old children exhibit a “brilliance = males” stereotype and whether parental attitudes toward gender roles were related to children’s stereotypes. We also explored whether the children exhibited such stereotypes in response to various stimuli. We showed children photos (Study 1) and stick figures (Study 2) of men, women, boys, and girls, asking them to attribute traits (smart or nice) to each. Study 1 revealed overwhelming in-group positivity in girls, whereas the results for boys were rather mixed. In Study 2, girls generally attributed nice to their own gender compared to boys. However, “brilliance = males” stereotypical responses were observed from 7 years of age, when boys began to be more likely to attribute smartness to their own gender compared to girls. The new data in Study 3 replicated results of Study 1 and parts of the results of Study 2. Moreover, merging the Study 3 data with that of Studies 1 and 2 confirmed their findings. Furthermore, it replicated the “brilliance = males” stereotype among 7-year-olds in the stick figure task. Parental attitudes toward gender roles were unrelated to children’s gender stereotypes. The results indicated that Japanese children may acquire “brilliance = males” stereotypes later than American children (6-years-old). Furthermore, the results were clearer when children were presented with stick figure stimuli.

## Introduction

Although many nations have been committed to promoting gender equality in the last several decades, the presence of a gender gap wherein women are less likely to participate in society, such as in political, educational, academic, or economic areas, remains one of the biggest issues in the twenty-first century. A typical gender gap has also been observed in scientific communities: fewer women work in certain professional areas than men, and fewer women authors appear in science, technology, engineering, mathematics, and medicine (STEMM) areas^1^. These areas are traditionally believed to be for men, and people’s beliefs, such as “men are better at science than women,” are examples of a gender stereotype that does not reflect the truth^2^.

Developmental psychologists have been interested in the origins of gender stereotypes. Previous studies have reported that people, including school-age children and college students, are generally more likely to believe that math and computers are for boys and men than for girls and women^3–6^. Parents and teachers in elementary schools are also likely to believe that boys are better at math and science^6–9^. Such adult beliefs could affect children’s attitude toward math^10^ (see also the review in Gunderson et al.^11^), and school-aged American and Singaporean children, as early as grades two or three, showed both implicit and explicit gender stereotypes such as “math is for boys”^3,4^. Moreover, Miller et al.^12^ reported that American k-12 children were more likely to draw a man when they were told to draw a scientist. Although fewer children have shown this tendency during the last five decades, they were still more likely to draw a male scientist as they grew older^12^. Similarly, 6- to 10-year-old children tended to draw a man as an adult mathematician, and this tendency was more obvious in boys; only girls drew a girl when they were asked to draw a child mathematician^13^. Children may believe that women, but not girls, are not good at mathematics^13^. Girls may acquire such gender stereotypes with increasing age, and such stereotypes could limit women of all ages^14^. That is, a girl could lose her interest in being a mathematician or scientist or give up on it, even when she is capable; falsely believing that it is a man’s job.

Gender stereotypes are not limited to a particular field that requires logical reasoning abilities, such as math, science, and computers. People are also likely to associate more fundamental abilities, such as intellectual ability (e.g., brilliance and genius), with men/boys^14,15^. This tendency was robustly observed in adults living in 78 countries in Europe, Asia, Latin America, the Caribbean, and Africa^16^. A previous study reported that the “brilliance = males” stereotype emerged among children as early as 6 years of age in the U.S. and a similar developmental pattern was observed with the “nice = female” stereotype^14^. Additionally, American 5- to 7-year-old children were less likely to choose girls as teammates for a game designed for really smart children^15^ and 6- to 7-year-old girls were less likely to be interested in such a game^14^.

Therefore, three issues must be explored in the present study. First, it is important to investigate whether the “brilliance = males” stereotype in children is observed in a country with a larger gender gap. In 2021, the World Economic Forum reported that the gender gap (regarding the general gender balance of four categories: economic participation and opportunity, educational attainment, health and survival, and political empowerment) in Japan was ranked 120th, while the United States was ranked 30th out of 156 countries. Previous studies have indicated that the level of the national gender gap could influence gender stereotypes. For example, implicit national gender-science stereotypes such as “math/science for males” predicted 8th-grade children’s mathematics and science performance in 34 countries^17^. Similar results were also reported for math and reading abilities in 15-year-olds from 40 countries^18^. Moreover, women’s participation rate in science (i.e., enrollment of tertiary science education and employment in researcher workforce) has also been related to explicit and/or implicit gender-science stereotypes in a study of 66 countries^19^. As noted above, Holman et al.^1^ explored academic authors within STEMM areas’ genders from 2002 to 2017, and reported that the percentage of women authors in Japan was the lowest (20.4%). Therefore, Japan is a good sample for exploring the prevalence of children’s “brilliance = males” stereotypes in a society with a large gender gap. In this study, we surveyed 4- to 7-year-old Japanese children.

Second, it is important to investigate whether “brilliance = males” stereotypes are exhibited as a result of various stimuli. Bian et al.^14^ used photo stimuli of men/boys and women/girls that controlled for attractiveness and clothes; however, they did not control for colors. It is well known that some colors, such as pink or blue, are considered gender-stereotyped colors^20–22^. Moreover, one study reported that blue could be associated with competence^23^. Photo stimuli could also include other rich perceptual cues such as faces, hairstyles, body shapes, colors, and so on. Such additional information, rather than the concept of gender, could have influenced children’s belief in the “brilliance = males” stereotype in Bian et al.’s study^14^. Therefore, it might be presumptuous to conclude that children are likely to associate men/boys with brilliance before conducting tests with a simpler stimulus. The present study used both photos with rich perceptual cues (Study 1) and simple stimuli, such as stick figures with fewer perceptual cues (Study 2). We also conducted an additional study, Study 3, to investigate whether there was any order effect because the same children first participated in Study 1 and then in Study 2. We also analyzed the merged data of Study 3 and Studies 1 and 2 to replicate their results in a larger sample.

Third, we examined factors related to individual differences in children’s gender stereotypes. Parents and teachers are likely to believe that sons (boys) are better at math and science^6–9^ or are brilliant^24^. Children learn gender stereotypes from their parents and can also develop their own gender stereotypes. In fact, previous studies have pointed out that parents’ and teachers’ beliefs, expectations, and gender stereotypes about boys being better at mathematics and science and/or girls being better at reading might relate to children’s perception of their ability in these subjects^6,25^ (see the review by Gunderson et al.^11^). Moreover, parents with stronger math-gender stereotypes were more likely to apply these beliefs to their own children, and parents’ beliefs about their children’s mathematical ability predicted their children’s self-perception regarding mathematics ability^10^. While some studies reported that such parental influence exists, others have reported that such influence depends on the subject. Mothers were less likely to be biased regarding who is better at mathematics than who is better at reading, and unlikely to reading, mothers’ beliefs about mathematics did not influence their children^6^. Moreover, some studies have reported that the gender of parent–child dyads is an important factor in how parental attitude influences children’s perception of their mathematics ability. For example, mothers’ gender-math stereotypes predicted their daughters’, but not their sons’ perception of their math ability^26^. Prevalence of gender-math stereotypes in mothers related to their daughters’ math performances under stereotype threat, or when making the girl participants’ gender identity salient by drawing a girl in a story that the participants had been told^27^. Given these findings, children could develop gender stereotypes based on parental beliefs. Therefore, the present study investigated whether Japanese parental attitudes toward gender roles are related to their children’s gender stereotypes.

We propose three hypotheses for this study: First, Japanese children in this large gender-gap society would show gender stereotypes for intellectual ability earlier than children in a smaller gender-gap society. Children in the U.S. showed such stereotypes at the age of six when asked to associate intellectual ability (i.e., smart) with one gender^14^, or even did so at the age of five when asked to choose a teammate to play a game designed for smart children^15^. Given the larger gender gap in Japan (120th) compared to that in the U.S. (30th), we predicted that Japanese children would show gender stereotypes slightly earlier than the American children in Bian et al.’s study^14^. More importantly, this study investigated how perceptual cues influence children’s gender stereotypes. Therefore, as the second hypothesis, we expected that children’s gender stereotypes would be weaker in Study 2 than in Study 1, assuming that rich perceptual cues would induce children’s gender stereotypes. Alternatively, children would similarly show gender stereotypes in both Studies 1 and 2 (and Study 3 conducted in a reversed order) if the concept of gender, but not additional perceptual cues such as colors, induces children’s gender stereotypes. Third, we hypothesized that parental beliefs about gender roles are related to their children’s gender stereotypes. Specifically, if parents have a less egalitarian attitude toward gender roles, their children are more likely to exhibit gender stereotypes.

## Study 1

We used a colored photo stimulus in Study 1. For both studies 1 and 2, we preregistered our hypotheses, method, primary analyses, and sample size (https://osf.io/h2p5y).

### Method

#### Participants

We determined the sample size based on the results in Study 2 in Bian et al.^14^. The study included 48 children (24 girls and 24 boys) in each age group, and excluded children who failed the screening questions (see below). We recruited 60 participants (30 girls and 30 boys) from each age group because it was difficult to determine the number of children who would pass the screening questions beforehand. The previous study included 5-, 6-, and 7-year-old children; however, we expected that Japanese children would show gender stereotypes earlier than American children^14^; therefore, we randomly recruited 240 4, 5-, 6-, and 7-year-old children (30 girls and 30 boys in each age group) and their parents from a database (Cross Marketing Inc. Tokyo, Japan, which is a crowd-sourcing company similar to Amazon Mechanical Turk). First, we asked parents in the database to determine whether they were interested in the research. We then gave the parents details about the research. If parents and children agreed to participate in the study, they were recruited. Twenty children (one 4-year-old, two 5-year-old, two 6-year-old, and five 7-year-old girls, and three 4-year-old, three 5-year-old, two 6-year-old, and two 7-year-old boys) did not pass the screening questions for either one of the two traits (smart or nice); these children were excluded from the analyses. The characteristics of the final sample are listed in Table 1. There were no significant differences in the months between the girls and boys (*p* = 0.339). None of the children had any known developmental abnormalities. Informed consent was obtained from all parents prior to their child’s involvement in the study, which was conducted in accordance with the principles of the Declaration of Helsinki and was approved by the Ethics Committee of the Psychological Science Unit, Kyoto University.

**Table 1:** Final sample characteristics in Study 1 and 2.

Age	Girl		Boy	
	N	Mean age (SD)	N	Mean age (SD)
4	29	54.66 (3.73)	27	54.67 (3.36)
5	28	66.89 (3.01)	27	65.56 (3.37)
6	28	77.18 (3.32)	28	76.5 (3.13)
7	25	89.28 (3.30)	28	90.00 (3.71)

#### Measures

##### Gender stereotype task

We used a task of Study 2 in Bian et al.^14^ with some modifications. Each child was given a set of six screening questions designed to test whether they understood the meanings of the key terms “smart” (3 questions) and “nice” (3 questions). We used the term “smart” in the present study because a previous study used it as a child-friendly synonym for brilliance^14^. For each question, the parent described a hypothetical child’s behavior orally (e.g., “A child learns things fast”) and asked their child whether the relevant trait term could be applied to this child (e.g., “Is this child smart, or not smart?”). We used an a priori exclusion criterion of 2/3 for each trait.

As noted above, photo stimuli for Japanese women and men, and girls and boys were used in Study 1. Preliminarily, 40 Japanese adults (20 women and 20 men) rated attractiveness (“How attractive does this person look?”) for both adult and child photographs and clothing (“How professionally is this person dressed?”) for adult photos on a seven-point scale. We then used adult photo stimuli that were matched in terms of attractiveness and type of clothing (i.e., how professionally dressed) and child photos that were matched in attractiveness in the main experiment.

There were two tasks. Task (i) included four stories, each of which described a hypothetical adult (child) whose gender was left unspecified. Two stories were about adults, and two were about children. The stories were about a “really, really smart” person (child), and a “really, really nice” person (child). After the parent told the child one of the stories, the child was shown four pictures in a line (two women and two men or two girls and two boys) and was asked to guess which one of the four adults (children) might be the person (child) in the story.

In Task (ii), the child was shown eight webpages individually. On each page, there were two individuals in the photos. For the first four trials, the pictures included a woman and a man. For the next four trials, the pictures included a girl and boy. The parent told the child that one of the two people was “really, really smart” (on 4 out of 8 trials) or “really, really nice” (in the other trials) and asked the child to guess which of the two had the relevant trait. The order of the pictures was pseudo-random.

In both tasks, a child received a score of 1 for the trial when he/she chose an adult (child) of the same gender as themselves (e.g., if a girl picked a woman); otherwise, they received a score of zero. The scores ranged from 0 to 6 for both the smart and nice traits. Higher stereotype scores indicated that the children associated each trait with their own gender (for example, higher smart stereotype scores in boys indicated that boys associated being “smart” with men/boys).

##### Gender role questionnaire

Parents were provided with the Japanese version of a questionnaire that examined their beliefs about gender roles^28^ on a five-point scale. This was a single-factor questionnaire that assessed one’s attitude toward gender roles, such as marital relationships (e.g., “A husband should decide on important matters in marriage”). We used the total questionnaire scores for the analyses. Higher scores indicate that parents have stronger beliefs about gender roles.

#### Procedure

We conducted the experiments online. Participants were first given gender stereotype tasks and then provided with a gender role questionnaire. First, a parent asked his or her child questions based on written instructions in an online form. To prevent the parents from using their own words to instruct their child, we dictated that the parents read the instructions directly, which minimized their influence. Second, the parent responded to the gender role questionnaire.

### Analytic plan

We used both adult and child stimuli and did not find any differences in the scores between adult and child stimuli. Thus, based on a previous study, we combined participants’ responses to adult and child stimuli^14^.

Children’s stereotype scores (combined across the two tasks) were submitted to a multilevel mixed-effects linear model with trait (smart vs. nice; level-1 predictor), gender (boys vs. girls; level-2 predictor), and age (4–5-, 6- vs. 7-year-olds; level-2 predictor), and all possible interaction terms were set as categorical fixed effects and a random intercept for participants. In addition, we included parents’ gender (mother vs. father) as a categorical fixed effect to control for this effect. Restricted maximum likelihood (REML) was used for model fitting. Type II sum of squares was used, and the degrees of freedom were determined using the Kenward-Roger approximation^29^. When an interaction term including children’s gender was significant, we conducted follow-up pairwise comparisons to evaluate differences in mean gender stereotype scores between boys and girls within each age and trait. Moreover, although we did not register, we conducted exploratory one-sample *t*-tests to assess whether children’s gender stereotype scores differed significantly from chance (0.5) for each gender and age for each trait. The analyses can assess whether children responded randomly or have a gender stereotype by comparing the chance level. In follow-up analyses and exploratory one-sample *t*-tests, *p*-values were adjusted using Holm’s method^30^, and the family wise error rate was controlled at 0.05.

The second analysis assessed the correlations between children’s stereotype scores and parents’ beliefs about gender roles for each gender group.

All analyses were conducted in R 4.0.3 (R Core Team. 2020). We used *the lme4*^31^ and *lmerTest*^32^ packages for the model fitting. We used *the emmeans* package^33^ for follow-up analyses and exploratory one-sample *t*-tests. We used *simr* package^34^ to estimate simulation-based power.

### Results and discussion

**Figure 1:**
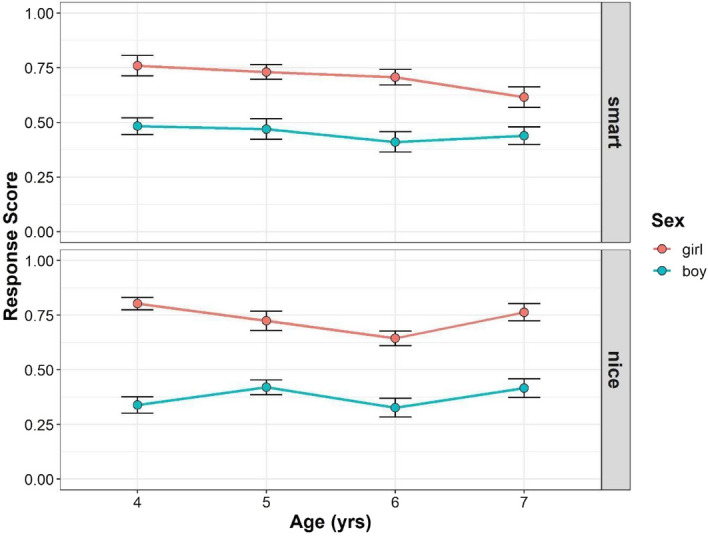
Developmental change of children’s response scores in Study 1. Boys’ (blue) and girls’ (red) mean scores (dots) are shown. Error bars represent ± 1 SE.

Figure 1 shows the developmental changes in the children’s response scores in Study 1 (see also Supplementary Tables S1, S2). To assess whether and how age, gender, and trait were related to the mean gender stereotype scores, we conducted multilevel mixed-effects linear modeling. For interaction terms, we found no significant effect of interaction between trait, gender, and age (*F* (3, 212) = 1.394, *p* = 0.246, simulation-based power = 0.399), and interaction between age and gender (*F* (3, 211) = 1.214, *p* = 0.305, power = 0.337), but we found significant interactions between trait and gender (*F* (1, 212) = 8.560, *p* = 0.004, power = 1), and trait and age (*F* (3, 212) = 2.713, *p* = 0.046, power = 0.642). For the main effects, we found a significant main effect of gender (*F* (1, 211) = 189.935, *p* < 0.001, power = 1) but the main effects of age (*F* (3, 211) = 2.306, *p* = 0.078, power = 0.592), and trait, (*F* (1, 212) = 1.792, *p* = 0.182, power = 0.254) were not significant. The main effect of parental gender was also not significant (*F* (1, 211) = 3.587, *p* = 0.060).

Follow-up pairwise comparisons were performed to compare the mean gender stereotype scores between boys and girls for each age and trait. In the smart traits, girls scored higher than boys in each age group (4-year-olds: girl–boy = 0.265, *t*(408) = 4.689, *p* < 0.001; 5-year-olds: girl–boy = 0.270, *t*(409) = 4.669, *p* < 0.001; 6-year-olds: girl–boy = 0.301, *t*(409) = 5.313, *p* < 0.001; 7-year-olds: girl–boy = 0.158, *t*(406) = 2.780, *p* = 0.006). In the nice traits, girls scored higher than boys in each age group (4-year-olds: girl–boy = 0.451, *t*(408) = 8.001, *p* < 0.001; 5-year-olds: girl–boy = 0.313, *t*(409) = 5.413, *p* < 0.001; 6-year-olds: girl–boy = 0.321, *t*(409) = 5.668, *p* < 0.001; 7-year-olds: girl–boy = 0.328, *t*(406) = 5.757, *p* < 0.001). In summary, although girls’ gender stereotype scores were higher than those of boys in both smart and nice traits, the difference between boys and girls was stronger in the nice traits than in the smart traits.

Moreover, we tested whether the children’s gender stereotype scores differed from chance for each gender and age for each trait. For girls’ gender stereotype scores in the smart traits, the 4- to 6-year-old children’s scores were significantly higher than the chance level, but the 7-year-old children’s scores were not (4-years-olds: *t*(383) = 5.072, *p* < 0.001; 5-year-olds: *t*(398) = 4.807, *p* < 0.001; 6-year-olds: *t*(392) = 4.319, *p* < 0.001; 7-year-olds: *t*(388) = 1.877, *p* = 0.221). For boys’ gender stereotype scores in the smart traits, the 6-year-old children’s scores were significantly lower than the chance level, but the 4-, 5-, and 7-year-old children’s scores were not (4-year-olds: *t*(398) = − 0.980, *p* = 0.328; 5-year-olds: *t*(387) = − 1.438, *p* = 0.303; 6-year-olds: *t*(388) = − 2.738, *p* = 0.045; 7-year-olds: *t*(405) = − 1.923, *p* = 0.221). For girls’ gender stereotype scores in the nice traits, the children’s scores were significantly higher than the chance level in all age groups (4-years-oldss: *t*(383) = 6.047, *p* < 0.001; 5-year-olds: *t*(398) = 4.658, *p* < 0.001; 6-year-olds: *t*(392) = 2.798, *p* = 0.043; 7-year-olds: *t*(388) = 5.195, *p* < 0.001). For the boys’ gender stereotype scores in the nice traits, the 4- and 6-year-old children’s scores were significantly lower than the chance level, but the 5- and 7-year-old children’s scores were not (4-year-olds: *t*(398) = − 4.507, *p* < 0.001; 5-year-olds: *t*(387) = − 2.567, *p* = 0.064; 6-year-olds: *t*(388) = − 4.613, *p* < 0.001; 7-year-olds: *t*(405) = − 2.494, *p* = 0.065).

Next, correlational analyses revealed no correlation between parental attitudes toward gender roles and boys’ mean gender stereotype scores in the smart (*r* = − 0.161, *p* = 0.078) and the nice (*r* = − 0.031, *p* = 0.731) traits. No relationships were found between parental attitudes and girls’ mean gender stereotype scores in both the smart (*r* = 0.047, *p* = 0.613) and the nice (*r* = 0.023, *p* = 0.798) traits.

Overall, girls’ scores were higher than those of boys for both the smart and nice traits. This was consistent with the overwhelming in-group positivity previously observed in young girls^35,36^. Four- to 6-year-old girls were more likely to attribute smartness to their own gender while 7-year-old girls were not. Only 6-year-old boys were less likely to attribute smartness to their own gender, while boys in other age groups were not. Girls in each of this study’s age groups were more likely to attribute being nice to their gender. Four- and 6-year-old boys were less likely to attribute nice to their own gender, while boys in other age groups were not. We did not find a relationship between parental attitudes toward gender roles and their children’s tendency to attribute smart or nice to their own gender.

In Study 1, we did not completely replicate the findings of Bian et al.’s study^14^. We used photo stimuli that included rich perceptual cues in Study 1. In Study 2, we used a simpler stimulus that eliminated the effects of additional information without eliminating the concept of gender.

## Study 2

We conducted Study 2 to explore whether “brilliance = males” stereotypes could be observed with simpler stimuli, such as stick figures in black and white.

### Participants

The same participants in Study 1 participated in Study 2.

### Procedure

Study 2 was conducted immediately after Study 1. The procedure was the same as in Study 1, except for three points. First, we used black and white stick figures instead of colored photo stimuli. Second, the children were given two questions about whether they understood the gender of stick figures (e.g., “Which figures represent a woman?”) prior to the experiment. One 4-year-old and two 5-year-old boys did not pass the questions and were excluded from the analyses. Finally, the children were given four trials instead of eight trials in task (ii) to reduce the task demand. For the first two trials, the stimuli included a woman and a man. For the next two trials, the stimuli included a girl and a boy. The parent told the child that one of the two people was “really, really smart” (2 out of 4 trials) or “really, really nice” (other trials) and asked the child to guess which of the two had the relevant trait. The scores ranged from 0 to 4 for both the smart and nice traits.

### Analytic plan

The analyses were the same as in Study 1.

### Results and discussion

**Figure 2:**
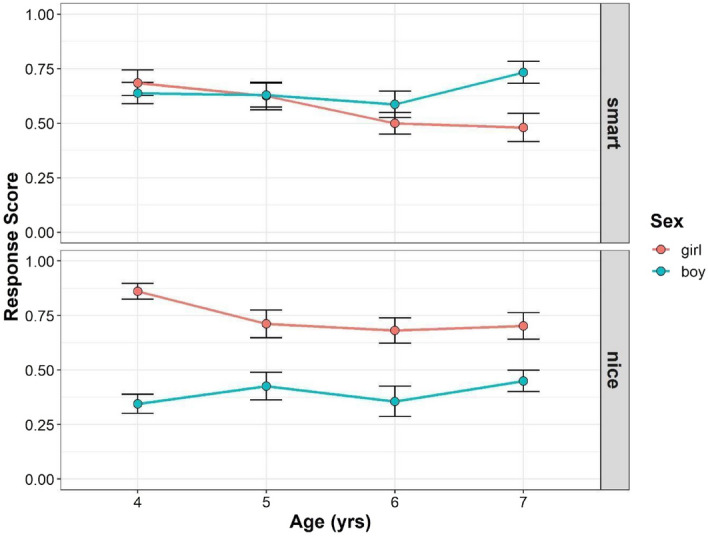
Developmental change of children’s response scores in Study 2. Boys’ (blue) and girls’ (red) mean scores (dots) are shown. Error bars represent ± 1 SE.

Figure 2 shows the developmental changes in children’s mean gender stereotype scores in Study 2 (see also Supplementary Tables S1, S3). For interaction terms, we found no significant effect of interaction among trait, gender, and age (*F* (3, 209) = 1.241, *p* = 0.295, power = 0.375), and between age and trait (*F* (3, 209) = 0.066, *p* = 0.978, power = 0.07). However, we found significant interactions between trait and gender (*F* (1, 209) = 67.347, *p* < 0.001, power = 1), and age and gender (*F* (3, 208) = 3.438, *p* = 0.018, power = 0.773). For the main effects, we found a significant main effect of gender (*F* (1, 208) = 18.237, *p* < 0.001, power = 0.993), but the main effects of age (*F* (3, 208) = 1.812, *p* = 0.146, power = 0.468), and trait (*F* (1, 209) = 2.472, *p* = 0.117, power = 0.290) were not significant. The main effect of the parents’ gender was also not significant (*F* (1, 208) = 0.002, *p* = 0.961).

Follow-up pairwise comparisons were performed to compare the mean gender stereotype scores between boys and girls for each age and trait. In the smart traits, there were no significant gender differences in gender stereotype scores in the 4-, 5-, and 6-year-old age groups, but the 7-year-old boys’ scores were significantly higher than those of the girls’ (4-year-olds: girl–boy = 0.052, *t*(395) = 0.645, *p* = 1.000; 5-year-olds: girl–boy = 0.015, *t*(395) = 0.177, *p* = 1.000; 6-year-olds: girl–boy = − 0.087, *t*(396) = − 1.086, *p* = 0.835; 7-year-olds: girl–boy = − 0.252, *t*(392) = − 3.133, *p* = 0.011). In the nice traits, girls scored higher than boys in each age group (4-year-olds: girl–boy = 0.513, *t*(395) = 6.411, *p* < 0.001; 5-year-olds: girl–boy = 0.261, *t*(395) = 3.148, *p* = 0.011; 6-year-olds: girl–boy = 0.325, *t*(396) = 4.070, *p* < 0.001; 7-year-olds: girl–boy = 0.253, *t*(392) = 3.142, *p* = 0.011). To summarize, girls’ gender stereotype scores were generally higher than boys in the nice trait, but there were no such trends in the smart traits.

Moreover, we tested whether the children’s gender stereotype scores differed from chance level for each gender and age, for each trait. For girls’ gender stereotype scores in the smart traits, the 4-year-old children’s scores were significantly higher than the chance level, but the 5-, 6-, and 7-year-old children’s scores were not (4-years-olds: *t*(367) = 2.967, *p* = 0.035; 5-year-olds: *t*(382) = 2.069, *p* = 0.274; 6-year-olds: *t*(376) = 0.018, *p* = 1.000; 7-year-olds: *t*(372) = − 0.286, *p* = 1.000). For boys’ gender stereotype scores in the smart traits, the 7-year-old children’s scores were significantly higher than the chance level, but the 4-, 5-, and 6-year-old children’s scores were not (4-year-olds: *t*(380) = 2.285, *p* = 0.206; 5-year-olds: *t*(373) = 1.737, *p* = 0.500; 6-year-olds: *t*(372) = 1.393, *p* = 0.823; 7-year-olds: *t*(391) = 4.252, *p* < 0.001). For girls’ gender stereotype scores in the nice traits, the children’s scores were significantly higher than the chance level in all age groups (4-years-olds: *t*(367) = 5.766, *p* < 0.001; 5-year-olds: *t*(382) = 3.492, *p* = 0.008; 6-year-olds: *t*(376) = 3.095, *p* = 0.025; 7-year-olds: *t*(372) = 3.224, *p* = 0.018). For the boys’ gender stereotype scores in the nice traits, the children’s scores in all age groups were not significantly different from the chance level (4-year-olds: *t*(380) = − 2.556, *p* = 0.110; 5-year-olds: *t*(373) = − 0.762, *p* = 1.000; 6-year-olds: *t*(372) = − 2.270, *p* = 0.206; 7-year-olds: *t*(391) = − 0.900, *p* = 1.000).

Correlational analyses revealed no correlation between parental attitudes toward gender roles and boys’ scores in the smart (*r* = − 0.0057, *p* = 0.531) and nice traits (*r* = − 0.114, *p* = 0.215), and the girls’ scores in the smart (*r* = − 0.043, *p* = 0.636) and nice traits (*r* = 0.098, *p* = 0.280).

In Study 2, there was no gender difference in the 4- to 6-year-old children’s scores for the smart traits while the 7-year-old boys’ score for the smart traits was higher than that of the girls. Girls' overwhelming in-group positivity regarding smart traits disappeared at 5 years of age, while boys' overwhelming in-group positivity regarding smart traits emerged at 7 years of age. Again, we did not find a relationship between parental attitudes toward gender roles and their children’s tendency to attribute smartness or niceness to their own gender.

One potential factor contributing to the different results between Studies 1 and 2 might be the order of the stimulus. In these studies, the same children participated in both the photo stimuli task (Study 1) and the stick figure task (Study 2), and all children judged the photo stimulus first and the stick figure stimulus next. Thus, the photo stimuli task may have worked as a practice for the stick figure task. That is, the observed “brilliance = males” stereotypical belief in the stick figure task, but not the photo stimuli task, might be due to practice effects rather than the effects of perceptual cues. To address this issue, we recruited another group of children and performed the stick figure task first, followed by the photo stimuli task.

## Study 3

Study 3 examined whether the same results as in Study 1 and 2 were observed when children performed the stick figure task first, followed by the photo stimuli task. Specifically, we collected additional data with another group of children using the tasks presented in the reverse order of the previous studies. Moreover, Bian et al.^14^ reported that the observed gender stereotype effects were more apparent in a larger sample size. Thus, we analyzed the merged data of Studies 1, 2, and 3 while controlling order effects.

### Participants

We randomly recruited 100 participants (50 girls and 50 boys) and their parents, in each age group, from a database (Cross Marketing Inc. Tokyo, Japan). A total of 38 children (seven 4-year-old, two 5-year-old, five 6-year-old, and one 7-year-old girls, and six 4-year-old, nine 5-year-old, four 6-year-old, and four 7-year-old boys) did not pass the screening questions for either one of the two traits (smart or nice); these children were excluded from the analyses. Moreover, a total of 17 children (three 4-year-old, two 5-year-old, one 6-year-old girls, and three 4-year-old, four 5-year-old, one 6-year-old, and three 7-year-old boys) did not pass the screening questions for the stick figure. The characteristics of the final sample are listed in Table 2. There were no significant differences in the months between the girls and boys in the merged data (*p* = 0.272). None of the children had any known developmental abnormalities. Informed consent was obtained as in Studies 1 and 2.

**Table 2:** Final sample characteristics in Study 3.

Age	Girl		Boy	
	N	Mean age (SD)	N	Mean age (SD)
4	40	53.15 (3.07)	41	54.29 (3.36)
5	46	65.00 (3.30)	37	66.27 (3.53)
6	44	77.18 (3.34)	45	78.24 (3.16)
7	49	89.00 (2.76)	43	88.37 (3.23)

### Procedure

The procedure was the same as that in Studies 1 and 2, except for one point. That is, children participated in the stick figure task first, followed by the photo task.

### Analytic plan

The analyses were the same as in Studies 1 and 2. The analyses were conducted separately for the photo stimuli task and the stick figure task.

### Results and discussion

#### Photo stimuli task

**Figure 3:**
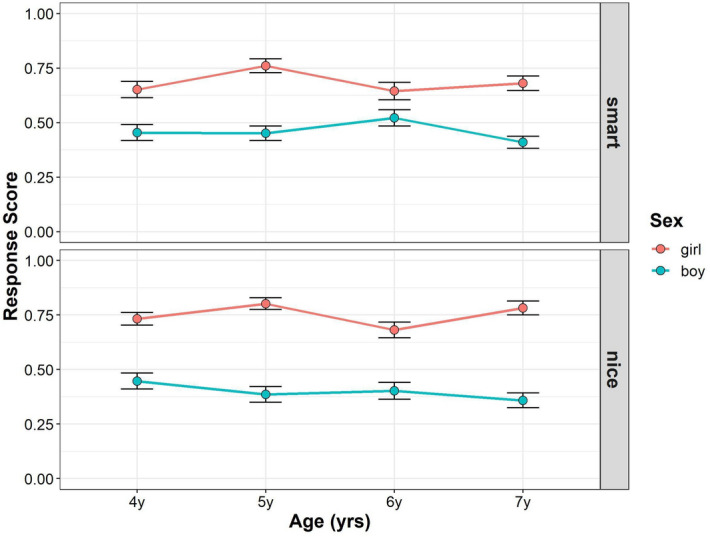
Developmental change of children’s response scores in photo stimuli task in Study 3. Boys’ (blue) and girls’ (red) mean scores (dots) are shown. Error bars represent ± 1 SE.

Figure 3 shows the developmental changes in children’s response scores in the photo stimuli task in Study 3 (see also Supplementary Tables S4, S5). To assess whether and how age, gender, and trait were related to the mean gender stereotype scores, we conducted multilevel mixed-effects linear modeling. For interaction terms, we found no significant effect of interaction among trait, gender, and age (*F* (3, 4354) = 0.372, *p* = 0.773, power = 0.140) and between age and trait (*F* (3, 354) = 1.722, *p* = 0.162, power = 0.457), but we found significant interactions between trait and gender (*F* (1, 354) = 21.378, *p* < 0.001, power = 0.996), and age and gender (*F* (3, 353) = 4.01, *p* = 0.008, power = 0.838). For the main effects, we found significant main effect of gender (*F* (1, 353) = 208.401, *p* < 0.001, power = 1), but the main effects of age (*F* (3, 353) = 1.061, *p* = 0.366, power = 0.258), and trait (*F* (1, 354) = 0.072, *p* = 0.789, power = 0.055), were not significant. The main effect of the parents’ gender (*F* (1, 353) = 1.148, *p* = 0.285) was also not significant.

Follow-up pairwise comparisons were performed to compare the mean gender stereotype scores between the boys and girls for each age and trait. In the smart traits, girls scored higher than boys in each age group (4-year-olds: girl–boy = 0.198, *t*_(625)_ = 3.997, *p* < 0.001; 5-year-olds: girl–boy = 0.308, *t*_(625)_ = 6.278, *p* < 0.001; 6-year-olds: girl–boy = 0.122, *t*_(625)_ = 2.518, *p* = 0.001; 7-year-olds: girl–boy = 0.275, *t*_(623)_ = 5.785, *p* < 0.001). In the nice traits, girls scored higher than boys in each age group (4-year-olds: girl–boy = 0.287, *t*_(625)_ = 5.794, *p* < 0.001; 5-year-olds: girl–boy = 0.415, *t*_(625)_ = 8.451, *p* < 0.001; 6-year-olds: girl–boy = 0.279, *t*_(625)_ = 5.753, *p* < 0.001; 7-year-olds: girl–boy = 0.428, *t*_(623)_ = 8.996, *p* < 0.001). Although girls’ gender stereotype scores were higher than those of boys in both the smart and nice traits, the difference between boys and girls was stronger in the nice traits than smart traits.

Moreover, we tested whether the children’s gender stereotype scores differed from chance level at each gender and age for each trait. For girls’ gender stereotype scores in the smart traits, the children’s scores were significantly higher than the chance level in all age groups (4-year-olds: *t*_(597)_ = 4.408, *p* < 0.001; 5-year-olds: *t*_(599)_ = 7.751, *p* < 0.001; 6-year-olds: *t*_(598)_ = 4.319, *p* < 0.001; 7-year-olds: *t*_(600)_ = 5.531, *p* < 0.001). For boys’ gender stereotype scores in the smart traits, the 7-year-old children’s scores were marginally lower than the chance level, but the 4-, 5-, and 6-year-old children’s scores were not (4-year-olds: *t*_(602)_ = − 0.930, *p* = 1; 5-year-olds: *t*_(599)_ = − 0.947, *p* = 1; 6-year-olds: *t*_(595)_ = 0.964, *p* = 1; 7-year-olds: *t*_(616)_ = − 2.414, *p* = 0.096). For girls’ gender stereotype scores in the nice traits, the children’s scores were significantly higher than the chance level in all age groups (4-year-olds: *t*_(597)_ = 6.595, *p* < 0.001; 5-year-olds: *t*_(599)_ = 8.936, *p* < 0.001; 6-year-olds: *t*_(598)_ = 5.339, *p* < 0.001; 7-year-olds: *t*_(600)_ = 8.476, *p* < 0.001). For boys’ gender stereotype scores in the nice traits, the children’s scores were significantly or marginally lower than the chance level in 5-, 6-, and 7-year-olds, but not 4-year-olds (4-year-olds: *t*_(602)_ = − 1.137, *p* = 1; 5-year-olds: *t*_(599)_ = − 2.659, *p* = 0.056; 6-year-olds: *t*_(595)_ = − 2.340, *p* = 0.098; 7-year-olds: *t*_(616)_ = − 3.878, *p* < 0.001). Thus, the results in Study 3 replicated the results in Study 1 in terms of girls’ scores were higher than those of boys for both the smart and nice traits.

#### Stick figure task

**Figure 4:**
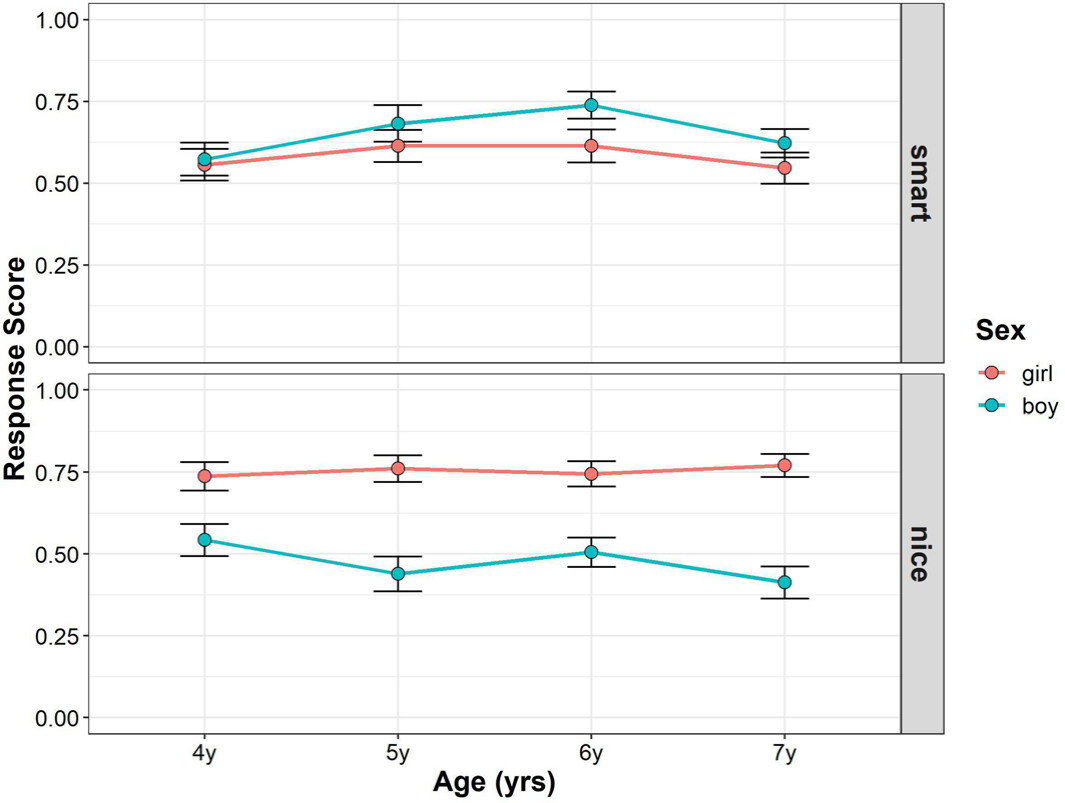
Developmental change of children’s response scores in stick figure task in Study 3. Boys’ (blue) and girls’ (red) mean scores (dots) are shown. Error bars represent ± 1 SE.

Figure 4 shows the developmental changes in the children’s response scores in the stick figure task in Study 3 (see also Supplementary Tables S4, S6). To assess whether and how age, gender, and trait were related to mean gender stereotype scores, we conducted multilevel mixed-effects linear modeling. For the interaction terms, we found no significant effect of interaction among trait, gender, and age (*F* (3, 337) = 1.418, *p* = 0.238, power = 0.385), interaction between age and gender (*F* (3, 336) = 0.565, *p* = 0.639, power = 0.194), and trait and age (*F* (3, 337) = 2.155, *p* = 0.093, power = 0.575), but we found a significant interaction between trait and gender (*F* (1, 337) = 77.898, *p* < 0.001, power = 1). For the main effects, we found significant main effect of gender (*F* (1, 336) = 16.021, *p* < 0.001, power = 1), but the main effects of age (*F* (3, 336) = 1.176, *p* = 0.319, power = 0.329), and trait (*F* (1, 337) = 0.021, *p* = 0.885, power = 0.044), were not significant. The main effects of parents’ gender (*F* (1, 336) = 0.186, *p* = 0.667) was also not significant.

Follow-up pairwise comparisons were performed to compare the mean gender stereotype scores between boys and girls for each age and trait. In the smart traits, there was no significant gender difference in gender stereotype scores in each age group. (4-year-olds: girl–boy = − 0.016, *t*_(632)_ = − 0.239, *p* = 0.811; 5-year-olds: girl–boy = − 0.070, *t*_(632)_ = − 1.041, *p* = 0.720; 6-year-olds: girl–boy = − 0.126, *t*_(632)_ = − 1.956, *p* = 0.204; 7-year-olds: girl–boy = − 0.075, *t*_(632)_ = − 1.176, *p* = 0.720). In the nice traits, girls scored higher than boys in each age group (4-year-olds: girl–boy = 0.196, *t*_(632)_ = 2.904, *p* = 0.019; 5-year-olds: girl–boy = 0.320, *t*_(632)_ = 4.779, *p* < 0.001; 6-year-olds: girl–boy = 0.238, *t*_(632)_ = 3.709, *p* = 0.001; 7-year-olds: girl–boy = 0.359, *t*_(632)_ = 5.663, *p* < 0.001). In summary, girls’ gender stereotype scores were generally higher than boys in the nice traits, but there were no such trends in the smart traits.

Moreover, we tested whether the children’s gender stereotype scores differed from chance level for each gender and age for each trait. For the girls’ gender stereotype scores in the smart traits, the 5-year-old children’s scores were marginally higher than the chance level, but the scores of the other children were not different from the chance level (4-year-olds: *t*_(603)_ = 1.251, *p* = 1; 5-year-olds: *t*_(604)_ = 2.550, *p* = 0.099; 6-year-olds: *t*_(606)_ = 2.492, *p* = 0.104; 7-year-olds: *t*_(607)_ = 1.143, *p* = 1). For boys’ gender stereotype scores in the smart traits, the 5-, 6-, and 7-year-old children’s scores were significantly or marginally higher than the chance level, but the 4-year-old children’s scores were not (4-year-olds: *t*_(608)_ = 1.603, *p* = 0.656; 5-year-olds: *t*_(599)_ = 3.569, *p* = 0.004; 6-year-olds: *t*_(602)_ = 5.106, *p* < 0.001; 7-year-olds: *t*_(620)_ = 2.670, *p* = 0.078). For girls’ gender stereotype scores in the nice traits, the children’s scores were significantly higher than the chance level in all age groups (4-year-olds: *t*_(603)_ = 4.806, *p* < 0.001; 5-year-olds: *t*_(604)_ = 5.642, *p* < 0.001; 6-year-olds: *t*_(606)_ = 5.195, *p* < 0.001; 7-year-olds: *t*_(607)_ = 6.056, *p* < 0.001). For the boys’ gender stereotype scores in the nice traits, the children’s scores in all age groups were not significantly different from chance (4-year-olds: *t*_(608)_ = 0.991, *p* = 1; 5-year-olds: *t*_(599)_ = − 0.982, *p* = 1; 6-year-olds: *t*_(602)_ = 0.266, *p* = 1; 7-year-olds: *t*_(620)_ = − 1.737, *p* = 0.580).

The results in Study 3 partially replicated those in Studies 1 and 2. Girls’ scores were higher than those of boys for nice traits. However, in terms of smart traits, boys’ scores were not significantly different from those of girls in each age group. These findings were inconsistent with Study 2, where boys’ scores were higher than that of girls in the 7-year-old age group but not in 4- to 6-year-old age groups. Nevertheless, the tendency was consistent in Study 2 in that older boys outperformed older girls. Given that the observed gender stereotype effects were evident in a larger sample size^14^, we merged the data of Study 3 with Studies 1 and 2 and analyzed the merged data when task order was included as a control variable.

#### Analyses of merged data

##### Photo stimuli task

**Figure 5:**
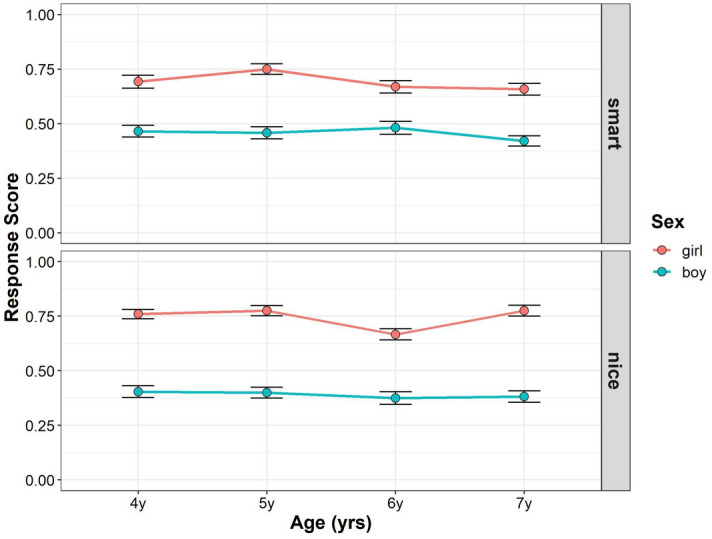
Developmental change of children’s response scores in photo stimuli task from Study1 and Study 3. Boys’ (blue) and girls’ (red) mean scores (dots) are shown. Error bars represent ± 1 SE.

Figure 5 shows the developmental changes in the children’s response scores in the photo stimuli figure task from Studies 1 and 3 (see also Supplementary Tables S7, S8). To assess whether and how age, gender, and trait were related to the mean gender stereotype scores, we conducted multilevel mixed-effects linear modeling. For interaction terms, we found no significant effect of interaction among trait, gender, and age (*F* (3, 574) = 0.528, *p* = 0.663, power = 0.193), and between age and gender (*F* (3, 572) = 1.799, *p* = 0.146, power = 0.487), but we found significant interactions between trait and gender (*F* (1, 574) = 29.418, *p* < 0.001, power = 0.998), and trait and age (*F* (3, 574) = 3.234, *p* = 0.022, power = 0.719). For the main effects, we found significant main effect of gender (*F* (1, 572) = 381.156, *p* < 0.001, power = 1), but the main effects of age (*F* (3, 572) = 2.089, *p* = 0.101, power = 0.540) and trait (*F* (1, 574) = 0.394, *p* = 0.531, power = 0.099) were not significant. The main effect of the parents’ gender (*F* (1, 572) = 0.006, *p* = 0.936) and task order (*F* (1, 572) = 0.209, *p* = 0.648) were also not significant.

Follow-up pairwise comparisons were performed to compare the mean gender stereotype scores between boys and girls for each age and trait. In the smart traits, girls scored higher than boys in each age group (4-year-olds: girl–boy = 0.227, *t*_(1045)_ = 6.032, *p* < 0.001; 5-year-olds: girl–boy = 0.291, *t*_(1046)_ = 7.724, *p* < 0.001; 6-year-olds: girl–boy = 0.188, *t*_(1046)_ = 5.051, *p* < 0.001; 7-year-olds: girl–boy = 0.236, *t*_(1041)_ = 6.416, *p* < 0.001). In the nice traits, girls scored higher than boys in each age group (4-year-olds: girl–boy = 0.355, *t*_(1045)_ = 9.443, *p* < 0.001; 5-year-olds: girl–boy = 0.375, *t*_(1046)_ = 9.939, *p* < 0.001; 6-year-olds: girl–boy = 0.292, *t*_(1046)_ = 7.855, *p* < 0.001; 7-year-olds: girl–boy = 0.393, *t*_(1041)_ = 10.691, *p* < 0.001). Although girls’ gender stereotype scores were higher than those of boys in both the smart and nice traits, the difference between boys and girls was larger in the nice than the smart traits.

Moreover, we tested whether the children’s gender stereotype scores differed from chance level for each gender and age for each trait. For girls’ gender stereotype scores in the smart traits, the children’s scores were significantly higher than the chance level in all age groups (4-year-olds: *t*_(987)_ = 6.636, *p* < 0.001; 5-year-olds: *t*_(1001)_ = 9.007, *p* < 0.001; 6-year-olds: *t*_(997)_ = 6.042, *p* < 0.001; 7-year-olds: *t*_(994)_ = 5.647, *p* < 0.001). For boys’ gender stereotype scores in the smart traits, the 7-year-old children’s scores were significantly lower than the chance level, but the 4-, 5-, and 6-year-old children’s scores were not (4-year-olds: *t*_(1007)_ = − 1.293, *p* = 0.412; 5-year-olds: *t*_(993)_ = − 1.488, *p* = 0.412; 6-year-olds: *t*(988) = − 0.715, *p* = 0.475; 7-year-olds: *t*_(1030)_ = − 3.049, *p* = 0.009). For girls’ gender stereotype scores in the nice traits, the children’s scores were significantly higher than the chance level in all age groups (4-year-olds: *t*_(987)_ = 8.949, *p* < 0.001; 5-year-olds: *t*_(1001)_ = 9.906, *p* < 0.001; 6-year-olds: *t*_(997)_ = 5.960, *p* < 0.001; 7-year-olds: *t*_(994)_ = 9.911, *p* < 0.001). For boys’ gender stereotype scores in the nice traits, the children’s scores were significantly lower than the chance level in all age groups (4-year-olds: *t*_(1007)_ = − 3.529, *p* = 0.003; 5-year-olds: *t*_(993)_ = − 3.514, *p* = 0.003; 6-year-olds: *t*_(988)_ = − 4.465, *p* < 0.001; 7-year-olds: *t*_(1030)_ = − 4.551, *p* < 0.001).

### Stick figure task

**Figure 6:**
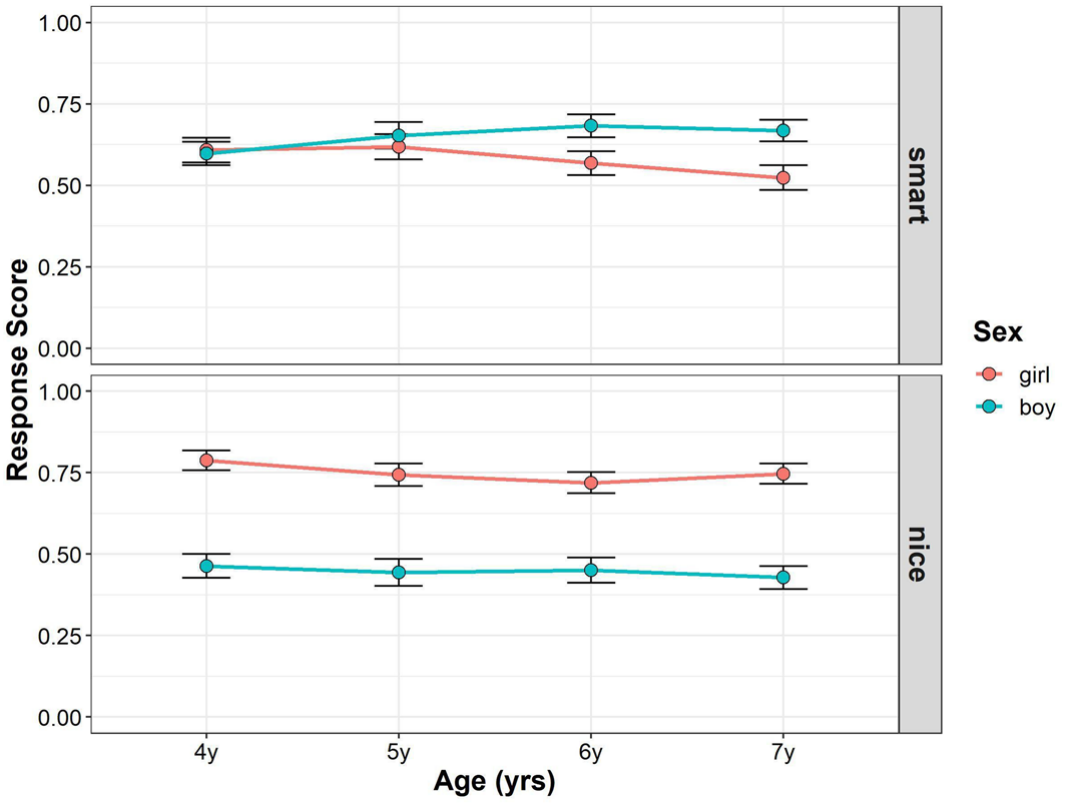
Developmental change of children’s response scores in stick figure task from Study1 and Study 3. Boys’ (blue) and girls’ (red) mean scores (dots) are shown. Error bars represent ± 1 SE.

Figure 6 shows the developmental changes in the children’s response scores in the stick figure task from Studies 2 and 3 (see also Supplementary Tables S7, S9). To assess whether and how age, gender, and trait were related to mean gender stereotype scores, we conducted multilevel mixed-effects linear modeling. For the interaction terms, we found no significant effect of interaction among trait, gender, and age (*F* (3, 554) = 1.153, *p* = 0.327, power = 0.309), interaction between age and gender (*F* (3, 552) = 1.069, *p* = 0.362, power = 0.304), and trait and age (*F* (3, 554) = 0.995, *p* = 0.395, power = 0.270), but we found a significant interaction between trait and gender (*F* (1, 554) = 144.696, *p* < 0.001, power = 1). For the main effects, we found significant main effect of gender (*F* (1, 552) = 32.185, *p* < 0.001, power = 1), but the main effects of age (*F* (3, 552) = 0.345, *p* = 0.793, power = 0.114) and trait (*F* (1, 554) = 0.731, *p* = 0.393, power = 0.132) were not significant. The main effects of parents’ gender (*F* (1, 552) = 0.192, *p* = 0.661) and task order (*F* (1, 552) = 1.803, *p* = 0.180) were also not significant.

Follow-up pairwise comparisons were performed to compare the mean gender stereotype scores between boys and girls for each age and trait. No significant gender difference in gender stereotype scores was found in the smart traits in 4-, 5-, and 6-year-olds, but the 7-year-old boys’ scores were significantly higher than the 7-year-old girls’ scores. (4-year-olds: girl–boy = 0.012, *t*(1040) = 0.222, *p* = 0.945; 5-year-olds: girl–boy = − 0.038, *t*(1039) = − 0.719, *p* = 0.945; 6-year-olds: girl–boy = − 0.114, *t*(1040) = − 2.271, *p* = 0.070; 7-year-olds: girl–boy = − 0.144, *t*(1037) = − 2.887, *p* = 0.016). In the nice traits, girls scored higher than boys in each age group (4-year-olds: girl–boy = 0.325, *t*(1040) = 6.262, *p* < 0.001; 5-year-olds: girl–boy = 0.297, *t*(1039) = 5.669, *p* < 0.001; 6-year-olds: girl–boy = 0.269, *t*(1040) = 5.340, *p* < 0.001; 7-year-olds: girl–boy = 0.319, *t*(1037) = 6.392, *p* < 0.001).

Moreover, we tested whether the children’s gender stereotype scores differed from chance level for each gender and age for each trait. For the girls’ gender stereotype scores in the smart traits, the 4- and 5-year-old children’s scores were significantly higher than the chance level, but the scores of the 6-and 7-year-old children were not (4-year-olds: *t*(981) = 2.813, *p* = 0.040; 5-year-olds: *t*(995) = 3.168, *p* = 0.014; 6-year-olds: *t*(992) = 1.893, *p* = 0.303; 7-year-olds: *t*(988) = 0.662, *p* = 0.819). For boys’ gender stereotype scores in the smart traits, the 5-, 6-, and 7-year-old children’s scores were significantly higher than the chance level, but the 4-year-old children’s scores were not (4-year-olds: *t*(999) = 2.621, *p* = 0.062; 5-year-olds: *t*(982) = 3.805, *p* = 0.001; 6-year-olds: *t*(983) = 4.808, *p* < 0.001; 7-year-olds: *t*(1021) = 4.657, *p* < 0.001). For girls’ gender stereotype scores in the nice traits, the children’s scores were significantly higher than the chance level in all age groups (4-years-olds: *t*(981) = 7.311, *p* < 0.001; 5-year-olds: *t*(995) = 6.479, *p* < 0.001; 6-year-olds: *t*(992) = 5.897, *p* < 0.001; 7-year-olds: *t*(988) = 6.650, *p* < 0.001). For the boys’ gender stereotype scores in the nice traits, the children’s scores in all age groups were not significantly different from chance (4-year-olds: *t*(999) = − 0.875, *p* = 0.819; 5-year-olds: *t*(982) = − 1.269, *p* = 0.819; 6-year-olds: *t*(983) = − 1.215, *p* = 0.819; 7-year-olds: *t*(1021) = − 1.958, *p* = 0.303).

The combined analyses showed that the girls’ scores were higher than those of boys for both the smart and nice traits in the photo stimuli task. They were more likely to attribute smartness to their own gender. Moreover, only 7-year-old boys were less likely to attribute smartness to their own gender, whereas boys in other age groups were not. Girls in each age group were more likely to attribute niceness to their own gender, and boys in each age group were less likely to attribute niceness to their own gender.

The results were consistent with those in Study 2 in the stick figure task. There was no gender difference in the 4- to 6-year-old children’s scores for the smart traits, while the 7-year-old boys’ scores for the smart traits were higher than those of the 7-year-old girls. Four and 5-year-old girls were more likely to attribute smartness to their own gender, whereas 6- and 7-year-old girls were not. Five to 7-year-old boys were more likely to attribute smartness to their own gender while 4-year-old boys were not. Girls in each age group were likely to attribute niceness to their own gender, while such attributions were at a chance level in boys in each age group.

## General discussion

In the present study, we investigated whether gender stereotypes regarding intellectual ability such as “brilliance (smart) = males” would be observed in 4- to 7-year-old Japanese children living in a society with a large gender gap. We tested children’s gender stereotypes about intellectual ability (with the child-appropriate word, “smart”) using photo stimuli with rich perceptual cues (e.g., faces, hairstyles, body shapes, and colors), which were also used in Study 1 in Bian et al.’s study^14^ and black and white stick figure stimuli with poor perceptual cues in Study 2. We conducted an additional study, Study 3, to replicate the results in Studies 1 and 2 even when the task order was reversed. We also examined whether parental attitudes toward gender roles were related to children’s “brilliance = males” stereotypes.

Partly in line with the previous study^14^ and the first hypothesis, the present results revealed that older Japanese children, approximately 7 years of age, may have gender stereotypes such as “brilliance = males.” However, there were minor differences between our results that used photo and stick figure stimuli and previous study’s^14^ results that used photo stimuli. Bian et al.^14^ reported gender differences in the scores for both the smart and nice traits in the older group (6- and 7-year-olds) but not in the younger group (5-year-olds). In the stick figure task (Study 2 and the merged data in Study 3) in the present study, multilevel mixed-effects linear modeling revealed that there were gender differences in gender stereotype scores among 7-year-olds in the smart traits: boys’ gender stereotype scores were higher than those of girls, whereas there was no gender difference among 4- to 6-year-olds. That is, girls’ tendency to attribute the smart trait to their gender shifted at 7 years of age, indicating “brilliance = males” stereotypes emerged at this age. Chance-level comparisons also supported this tendency. In the stick figure task, the older boys’ (7-year-olds in Study 2 and 5- to 7-year-olds in the merged data in Study 3) scores in the smart traits were significantly higher than the chance level, whereas such tendency was observed only in young girls (4-year-olds in Study 2 and 4- and 5-year-olds in the merged data in Study 3). Note that these results were not apparent when we analyzed Study 3 data alone. However, one of our purposes in conducting Study 3 was to replicate Studies 1 and 2 with larger samples and analyze the merged data. When we used photo stimuli (Study 1 and 3) similar to those used in Bian et al.^14^, these results were not apparent. Overall, girls showed higher scores than boys.

Moreover, Japanese girls’ scores for nice traits were higher than those of boys in both the photo and stick figure tasks. Unlike boys, girls in all age groups were more likely to attribute nice traits to their gender. They also showed higher scores for smart traits in the photo tasks. Previous studies have reported that girls are more likely to show in-group positivity than boys^14,15,35,36^. We assumed that Japanese girls were also likely to show this tendency. Finally, we did not find an order effect in the merged data of Study 3, possibly suggesting that the practice effect from the preceding task might not explain the difference between the photo and stick figure tasks in the present study. This could indicate that the nature of the stimuli is more important than the order of stimuli presentation.

Contrary to our hypothesis, Japanese children in a large gender gap society might show “brilliance = males” stereotypes later than American children in a small gender gap society. That is, the gender gap in adult contexts, such as “men are better at political, educational, or academic areas,” might not play a significant role in young children’s gender stereotypes regarding intellectual ability. We assumed that educational differences between Japan and the U.S. could be one of the possible factors explaining why Japanese children’s emergence of gender stereotypes occurred at a later stage. In the U.S., children generally start kindergartens that are connected to public schools at the age of five, and their curriculum is more like that of elementary schools, whereas such compulsory education begins in the first grade (at 6–7 years of age) in elementary schools in Japan^37^. Thus, children may acquire gender stereotypes about intellectual ability after they attend schools that teach subjects, including math and science, for compulsory education. This experience could explain why well-formed gender stereotypes were not observed in preschool children in the present study when analyzed with a smaller sample in Study 3. Indeed, an effect of schooling was observed in American girls by Bian et al.^14^.

Moreover, Japanese preschool teachers may not teach gender equality explicitly, but treat all children equally; therefore, preschool children might be less likely to form gender stereotypes. In fact, Japanese culture does not strictly control young people depending on gender; for example, young boys and girls, particularly around 9–10 years, are allowed to visit hot springs for both men and women with their parents. The gender gap might be one factor influencing people’s “brilliance = male” stereotypes, but it might be more important for adults than children. In fact, Steele^13^ reported that only 6- to 10-year-old girls drew a girl when asked to draw a child mathematician, while they drew a man for an adult mathematician, probably because girls believe that mathematics is for adult men, but when related to children’s issues, this is equal for boys and girls. There might be other factors leading to young children exhibiting gender stereotypes. Future studies need to explore detailed differences in gender education, how society treats gender differences, or children’s media exposure (e.g., how familiar are children with stories that show them boys are smart) in the two countries to clarify why there exists a cross-cultural difference between Japanese and American children.

Notably, the stick-figure task was more likely to elicit gender stereotypes than the photo task. This could be for two reasons. First, although the present study (and Bian et al.^14^) controlled for attractiveness and clothing in photo stimuli, the rich cues of individuals’ appearance in the photo stimuli (e.g., faces and hairstyles) might have weakened the ability to detect gender-stereotyped attitudes in children^38^. For instance, given that individuals’ hairstyles largely influence how they are perceived in terms of professionalism and competentence^39^, it is possible that participants’ evaluations here were influenced by the varied hairstyles of the actors (especially for women actors whose hairstyles ranged from short to long styles^40^).

Second, the differentiated effects of photo and stick figure stimuli on children’s evaluations might be derived from the distinction in the extent to which the stimuli could raise children’s awareness of gender categories. Given that photo stimuli include various cues (e.g., background, direct gaze), children may have used dimensions other than gender to categorize/discriminate the actors (e.g., categorizing the actors as trustworthy and untrustworthy individuals, but not as women/girls and men/boys). However, stick-figure stimuli may have a greater likelihood of being perceived and categorized according to gender. This is not only because the stick-figure stimuli provided no cues that could establish categories other than gender, but also because children were asked to identify the gender of the stick-figure stimuli before evaluating gender stereotypes, in which the gender concept in children may be emphasized. In short, compared to photo stimuli, stick figure stimuli might be more powerful in raising children’s awareness of gender categories. Previous studies have shown that 6- to 8-year-old children explicitly showed stereotyped attitudes toward individuals when they could be easily categorized into groups related to biased attitudes, but children do not do so when the target individuals belong to groups that differ along some dimensions but share commonalities distinguishing them from other groups^41,42^. This suggests that to elicit children’s explicit stereotyped attitudes, the stimuli may have to be particularly pronounced in raising children’s awareness of the groups related to the stereotypes. Therefore, it might be that the stick figure stimuli were efficient in raising children’s gender awareness, which in turn elicited a gender-stereotyped evaluation, but the photo stimuli did not. Here, we tested children’s explicit judgements (i.e., identifying the characters explicitly) regarding gender stereotypes. Compared with explicit evaluations, children’s implicit evaluations are more sensitive to less sharply-differentiated group differences^41,42^. In cases where children are asked to evaluate the properties of characters whose groups cannot be simply identified, measures of implicit evaluations (e.g., Implicit Association Test^43^) may be useful to detect children’s stereotyped beliefs.

Stick-figure stimuli may be appropriate for basic research because we can assess pure gender effects without perceptual cues. However, such stimuli may have few real-world applications. The photo stimuli are more directly related to real-world applications. Our results suggest that children may have gender stereotypes pertaining to brilliance (from the results in the stick figure task). However, children’s real-world stereotypes could be influenced by perceptual information, such as hair, facial features, and so on.

We did not find strong evidence supporting that parental attitude toward gender roles played a role in children’s gender stereotypes regarding intellectual ability. At least around the ages of four to seven, Japanese children might be less likely to acquire gender stereotypes according to their parents’ attitudes toward gender roles. Japanese parents may not talk about gender roles with their young children; they may treat boys and girls equally because it is too early to expect that their preschool children will have gender-stereotypical abilities, especially intellectual ones. It might be premature to conclude that parental attitudes do not relate to children’s gender stereotypes; therefore, further studies are needed to explore how parental attitudes toward gender roles affect children at later stages.

The present study has some limitations. We conducted an online survey because of the coronavirus pandemic. Parents instructed, showed stimuli, and questioned their children instead of trained experimenters; therefore, we cannot completely exclude the possibility that the children considered their parents’ reactions when they gave their responses. Moreover, we did not ask parents whether they believed men were more brilliant than women. To clarify parental influence, further studies are needed to examine how parents’ “brilliance = males” stereotypes predict their children’s same-gender stereotypes. Finally, a study reported that American children (i.e., White, Latinx, Asian, Black, and multiracial; however, the highest portion of participants were White) showed “brilliance = white males” gender stereotypes; however, they did not extend this tendency to black men: they were likely to rate black women as smarter than black men^44^. Although we found clearer results with stick figures than with photos, we used only pictures of Japanese adults in the photo task. The present data do not clarify whether Japanese children’s “brilliance = males” concepts are limited to those belonging to the same racial group or could be extended to individuals from other racial groups. Further studies involving Japanese children must include other races as stimuli to examine whether they show “brilliance = white males/same-race males” stereotypes.

This study has several important implications. A recent report revealed an obvious gender gap in academic sciences^1^. The present study found that the gender gap in adult contexts might not be important for young children to acquire gender stereotypes toward intellectual ability because they might acquire the gender stereotype “brilliance = males” after they go to school. Therefore, if early childhood education at schools is carefully conducted, children might be able to learn about gender equality.

## Supplementary Information

Supplementary Tables.
