# Supplementary material for: Gender stereotypes about intellectual ability in Japanese children
Source: Sci Rep. 2022 Oct 11;12:16748. doi: 10.1038/s41598-022-20815-2 (PMC9554173; doi:10.1038/s41598-022-20815-2)
Supplement: Supplementary file 1 — Supplementary Tables. [file 41598_2022_20815_MOESM1_ESM.docx]

**Gender stereotypes about intellectual ability in Japanese children**

Mako Okanda^1^, Xianwei Meng^2^, Yasuhiro Kanakogi^2^, Moe Uragami^3^, Hiroki Yamamoto^2,4^, Yusuke Moriguchi^4*^

1 Otemon Gakuin University

2 Graduate School of Human Sciences, Osaka University

3 Sugiyama Jogakuen University

4 Graduate School of Letters, Kyoto University

*Correspondence should be addressed to Yusuke Moriguchi, Graduate School of Letters, Kyoto University, Yoshidahoncho, Kyoto 606-8501, Japan

Tel: +81-75-753-2852; E-mail: moriguchi.yusuke.8s@kyoto-u.ac.jp

**Acknowledgements**

We thank the parents and children who participated in this study. We are also grateful to Dr. Makoto Mizusaki, Megumi Kuwabara, and Sawa Senzaki for their helpful comments.

**Supplementary Information**

**Supplementary Table S1. Boys’ and girls’ mean gender stereotype scores in Studies 1 and 2 (mean and standard deviation)**

| **Age** | **Gender** | **Study 1** | | **Study 2** | |
| --- | --- | --- | --- | --- | --- |
|  |  | **Smart** | **Nice** | **Smart** | **Nice** |
| **4-year-olds** | **Boys** | **0.48 (0.21)** | **0.34 (0.20)** | **0.63 (0.27)** | **0.35 (0.24)** |
|  | **Girls** | **0.76 (0.24)** | **0.80 (0.15)** | **0.69 (0.31)** | **0.86 (0.19)** |
| **5-year-olds** | **Boys** | **0.47 (0.24)** | **0.42 (0.18)** | **0.61 (0.29)** | **0.45 (0.33)** |
|  | **Girls** | **0.73 (0.17)** | **0.72 (0.23)** | **0.63 (0.33)** | **0.71 (0.32)** |
| **6-year-olds** | **Boys** | **0.41 (0.24)** | **0.33 (0.22)** | **0.59 (0.31)** | **0.36 (0.35)** |
|  | **Girls** | **0.71 (0.19)** | **0.64 (0.18)** | **0.50 (0.27)** | **0.68 (0.31)** |
| **7-year-olds** | **Boys** | **0.44 (0.22)** | **0.42 (0.23)** | **0.73 (0.28)** | **0.45 (0.27)** |
|  | **Girls** | **0.62 (0.24)** | **0.76 (0.20)** | **0.48 (0.33)** | **0.70 (0.31)** |

**Supplementary Table S2. Results of linear mixed model examining children’s gender stereotype scores in Study 1.**

| **Fixed Effects** | **Estimate** | **Std.Error** | **df** | **t** | **Pr (> \|t\| )** |
| --- | --- | --- | --- | --- | --- |
| **(Intercept)** | 0.690 | 0.054 | 324.32 | 12.674 | <0.001 |
| **age_5y** | -0.018 | 0.058 | 408.35 | -0.308 | 0.758 |
| **age_6y** | -0.045 | 0.056 | 408.95 | -0.804 | 0.422 |
| **age_7y** | -0.141 | 0.058 | 409.42 | -2.451 | 0.015 |
| **trait_nice** | 0.043 | 0.051 | 212.00 | 0.836 | 0.404 |
| **gender_boy** | -0.265 | 0.056 | 407.98 | -4.689 | <0.001 |
| **parent_mother** | 0.069 | 0.037 | 211.00 | 1.894 | 0.060 |
| **age_5y:trait_nice** | -0.050 | 0.074 | 212.00 | -0.672 | 0.502 |
| **age_6y:trait_nice** | -0.106 | 0.072 | 212.00 | -1.482 | 0.140 |
| **age_7y:trait_nice** | 0.104 | 0.074 | 212.00 | 1.412 | 0.160 |
| **age_5y:gender_boy** | -0.005 | 0.081 | 407.41 | -0.064 | 0.950 |
| **age_6y:gender_boy** | -0.036 | 0.080 | 408.07 | -0.456 | 0.648 |
| **age_7y:gender_boy** | 0.106 | 0.079 | 409.29 | 1.337 | 0.182 |
| **trait_nice:gender_boy** | -0.187 | 0.072 | 212.00 | -2.602 | 0.010 |
| **age_5y:trait_nice:gender_boy** | 0.144 | 0.103 | 212.00 | 1.397 | 0.164 |
| **age_6y:trait_nice:gender_boy** | 0.167 | 0.102 | 212.00 | 1.633 | 0.104 |
| **age_7y:trait_nice:gender_boy** | 0.017 | 0.102 | 212.00 | 0.169 | 0.866 |

**Supplementary Table S3. Results of linear mixed model examining children’s gender stereotype scores in Study 2**

| **Fixed Effects** | **Estimate** | **Std.Error** | **df** | **t** | **Pr (> \|t\| )** |
| --- | --- | --- | --- | --- | --- |
| **(Intercept)** | 0.688 | 0.078 | 308.34 | 8.804 | <0.001 |
| **age_5y** | -0.061 | 0.082 | 394.52 | -0.742 | 0.458 |
| **age_6y** | -0.185 | 0.079 | 395.25 | -2.341 | 0.020 |
| **age_7y** | -0.205 | 0.081 | 395.82 | -2.518 | 0.012 |
| **trait_nice** | 0.176 | 0.071 | 209.00 | 2.491 | 0.014 |
| **gender_boy** | -0.052 | 0.080 | 394.67 | -0.645 | 0.519 |
| **parent_mother** | -0.003 | 0.053 | 208.00 | -0.049 | 0.961 |
| **age_5y:trait_nice** | -0.089 | 0.101 | 209.00 | -0.886 | 0.376 |
| **age_6y:trait_nice** | 0.005 | 0.098 | 209.00 | 0.052 | 0.959 |
| **age_7y:trait_nice** | 0.045 | 0.101 | 209.00 | 0.448 | 0.654 |
| **age_5y:gender_boy** | 0.037 | 0.116 | 393.98 | 0.319 | 0.750 |
| **age_6y:gender_boy** | 0.138 | 0.113 | 394.60 | 1.221 | 0.223 |
| **age_7y:gender_boy** | 0.303 | 0.113 | 395.48 | 2.698 | 0.007 |
| **trait_nice:gender_boy** | -0.462 | 0.099 | 209.00 | -4.663 | <0.001 |
| **age_5y:trait_nice:gender_boy** | 0.215 | 0.143 | 209.00 | 1.507 | 0.133 |
| **age_6y:trait_nice:gender_boy** | 0.050 | 0.140 | 209.00 | 0.356 | 0.722 |
| **age_7y:trait_nice:gender_boy** | -0.043 | 0.140 | 209.00 | -0.307 | 0.759 |

**Supplementary Table S4. Boys’ and girls’ mean gender stereotype scores in photo stimuli task and stick figure task (mean and standard deviation) in Study 3**

| **Age** | **Gender** | **Photo stimuli task**  **(Study 3)** | | **Stick figure task**  **(Study 3)** | |
| --- | --- | --- | --- | --- | --- |
|  |  | **Smart** | **Nice** | **Smart** | **Nice** |
| **4-year-olds** | **Boys** | 0.46 (0.24) | 0.45 (0.24) | 0.57 (0.32) | 0.54 (0.32) |
|  | **Girls** | 0.65 (0.25) | 0.73 (0.19) | 0.56 (0.30) | 0.74 (0.28) |
| **5-year-olds** | **Boys** | 0.45 (0.22) | 0.39 (0.23) | 0.68 (0.34) | 0.44 (0.33) |
|  | **Girls** | 0.76 (0.22) | 0.80 (0.19) | 0.61 (0.33) | 0.76 (0.28) |
| **6-year-olds** | **Boys** | 0.52 (0.25) | 0.40 (0.26) | 0.74 (0.28) | 0.51 (0.30) |
|  | **Girls** | 0.64 (0.27) | 0.68 (0.24) | 0.61 (0.33) | 0.74 (0.26) |
| **7-year-olds** | **Boys** | 0.41 (0.19) | 0.36 (0.23) | 0.62 (0.29) | 0.41 (0.32) |
|  | **Girls** | 0.68 (0.23) | 0.78 (0.22) | 0.55 (0.33) | 0.77 (0.25) |

**Supplementary Table S5. Results of linear mixed model examining children’s gender stereotype scores in photo stimuli task in Study 3.**

| **Fixed Effects** | **Estimate** | **Std.Error** | **df** | **t** | **Pr (> \|t\| )** |
| --- | --- | --- | --- | --- | --- |
| **(Intercept)** | 0.680 | 0.044 | 520.94 | 15.338 | <0.001 |
| **age_5y** | 1.108 | 0.048 | 625.27 | 2.232 | 0.026 |
| **age_6y** | -0.007 | 0.049 | 625.34 | -0.148 | 0.882 |
| **age_7y** | 0.027 | 0.048 | 625.13 | 0.570 | 0.569 |
| **trait_nice** | 0.081 | 0.040 | 354.00 | 2.043 | 0.042 |
| **gender_boy** | -0.198 | 0.050 | 625.20 | -4.00 | <0.001 |
| **parent_mother** | -0.032 | 0.030 | 353.00 | -1.072 | 0.285 |
| **age_5y:trait_nice** | -0.040 | 0.055 | 354.00 | -0.724 | 0.469 |
| **age_6y:trait_nice** | -0.044 | 0.056 | 354.00 | -0.796 | 0.427 |
| **age_7y:trait_nice** | 0.021 | 0.055 | 354.00 | 0.378 | 0.706 |
| **age_5y:gender_boy** | -0.110 | 0.070 | 625.14 | -1.581 | 0.114 |
| **age_6y:gender_boy** | 0.076 | 0.070 | 625.16 | 1.098 | 0.273 |
| **age_7y:gender_boy** | -0.077 | 0.069 | 624.92 | -1.127 | 0.260 |
| **trait_nice:gender_boy** | -0.089 | 0.056 | 354.00 | -1.588 | 0.113 |
| **age_5y:trait_nice:gender_boy** | -0.018 | 0.080 | 354.00 | -0.225 | 0.822 |
| **age_6y:trait_nice:gender_boy** | -0.068 | 0.078 | 354.00 | -0.863 | 0.389 |
| **age_7y:trait_nice:gender_boy** | -0.064 | 0.078 | 354.00 | -0.823 | 0.411 |

**Supplementary Table S6. Results of linear mixed model examining children’s gender stereotype scores in stick figure task in Study 3.**

| **Fixed Effects** | **Estimate** | **Std.Error** | **df** | **t** | **Pr (> \|t\| )** |
| --- | --- | --- | --- | --- | --- |
| **(Intercept)** | 0.573 | 0.061 | 520.62 | 9.377 | <0.001 |
| **age_5y** | 0.057 | 0.066 | 632.20 | 0.874 | 0.382 |
| **age_6y** | 0.057 | 0.066 | 632.18 | 0.856 | 0.392 |
| **age_7y** | -0.012 | 0.065 | 631.89 | -0.178 | 0.858 |
| **trait_nice** | 0.181 | 0.059 | 337.00 | 3.093 | 0.002 |
| **gender_boy** | 0.016 | 0.067 | 632.12 | 0.239 | 0.811 |
| **parent_mother** | -0.018 | 0.041 | 336.00 | -0.431 | 0.667 |
| **age_5y:trait_nice** | -0.035 | 0.080 | 337.00 | -0.431 | 0.667 |
| **age_6y:trait_nice** | -0.051 | 0.081 | 337.00 | -0.625 | 0.533 |
| **age_7y:trait_nice** | 0.043 | 0.079 | 337.00 | 0.548 | 0.584 |
| **age_5y:gender_boy** | 0.054 | 0.100 | 631.61 | 0.564 | 0.573 |
| **age_6y:gender_boy** | 0.110 | 0.093 | 632.08 | 1.177 | 0.240 |
| **age_7y:gender_boy** | 0.059 | 0.092 | 632.22 | 0.633 | 0.527 |
| **trait_nice:gender_boy** | -0.212 | 0.082 | 337.00 | -2.571 | 0.011 |
| **age_5y:trait_nice:gender_boy** | -0.178 | 0.116 | 337.00 | -1.535 | 0.126 |
| **age_6y:trait_nice:gender_boy** | -0.152 | 0.114 | 337.00 | -1.338 | 0.182 |
| **age_7y:trait_nice:gender_boy** | -0.222 | 0.113 | 337.00 | -1.964 | 0.050 |

**Supplementary Table S7 Boys’ and girls’ mean gender stereotype scores in photo stimuli task and stick figure task (mean and standard deviation)**

| **Age** | **Gender** | **Photo stimuli task**  **(Study 1 & Study 3)** | | **Stick figure task**  **(Study 2 & Study 3)** | |
| --- | --- | --- | --- | --- | --- |
|  |  | **Smart** | **Nice** | **Smart** | **Nice** |
| **4-year-olds** | **Boys** | **0.47 (0.23)** | **0.40 (0.23)** | **0.60 (0.30)** | **0.46 (0.30)** |
|  | **Girls** | **0.69 (0.25)** | **0.76 (0.18)** | **0.61 (0.31)** | **0.79 (0.25)** |
| **5-year-olds** | **Boys** | **0.46 (0.23)** | **0.40 (0.21)** | **0.65 (0.32)** | **0.44 (0.32)** |
|  | **Girls** | **0.75 (0.21)** | **0.77 (0.20)** | **0.62 (0.33)** | **0.74 (0.29)** |
| **6-year-olds** | **Boys** | **0.48 (0.25)** | **0.38 (0.25)** | **0.68 (0.30)** | **0.45 (0.33)** |
|  | **Girls** | **0.67 (0.24)** | **0.67 (0.22)** | **0.57 (0.31)** | **0.72 (0.28)** |
| **7-year-olds** | **Boys** | **0.42 (0.20)** | **0.38 (0.23)** | **0.67 (0.29)** | **0.43 (0.30)** |
|  | **Girls** | **0.66 (0.24)** | **0.78 (0.21)** | **0.52 (0.33)** | **0.75 (0.27)** |

**Supplementary Table S8. Results of linear mixed model examining children’s gender stereotype scores in photo stimuli task from Study 1 and Study 3.**

| **Fixed Effects** | **Estimate** | **Std.Error** | **df** | **t** | **Pr (> \|t\| )** |
| --- | --- | --- | --- | --- | --- |
| **(Intercept)** | 0.680 | 0.043 | 743.6 | 15.695 | <0.001 |
| **age_5y** | 0.057 | 0.037 | 1045 | 1.522 | 0.128 |
| **age_6y** | -0.024 | 0.037 | 1046 | -0.636 | 0.525 |
| **age_7y** | -0.035 | 0.037 | 1046 | -0.945 | 0.345 |
| **trait_nice** | 0.067 | 0.032 | 574.0 | 2.110 | 0.035 |
| **gender_boy** | -0.227 | 0.037 | 1045 | -6.032 | <0.001 |
| **task order** | 0.007 | 0.016 | 572.0 | 0.457 | 0.648 |
| **parent_mother** | 0.002 | 0.023 | 572.0 | 0.080 | 0.936 |
| **age_5y:trait_nice** | -0.042 | 0.044 | 574.0 | -0.951 | 0.342 |
| **age_6y:trait_nice** | -0.069 | 0.044 | 574.0 | -1.564 | 0.118 |
| **age_7y:trait_nice** | 0.051 | 0.044 | 574.0 | 1.164 | 0.245 |
| **age_5y:gender_boy** | -0.065 | 0.053 | 1045 | -1.211 | 0.226 |
| **age_6y:gender_boy** | 0.039 | 0.053 | 1045 | 0.739 | 0.460 |
| **age_7y:gender_boy** | -0.009 | 0.052 | 1046 | -0.175 | 0.861 |
| **trait_nice:gender_boy** | -0.128 | 0.044 | 574.0 | -2.902 | 0.004 |
| **age_5y:trait_nice:gender_boy** | 0.047 | 0.063 | 574.0 | 0.714 | 0.476 |
| **age_6y:trait_nice:gender_boy** | 0.024 | 0.062 | 574.0 | 0.387 | 0.699 |
| **age_7y:trait_nice:gender_boy** | -0.029 | 0.062 | 574.0 | -0.469 | 0.639 |

**Supplementary Table S9. Results of linear mixed model examining children’s gender stereotype scores in stick figure task from Study 2 and Study 3.**

| **Fixed Effects** | **Estimate** | **Std.Error** | **df** | **t** | **Pr (> \|t\| )** |
| --- | --- | --- | --- | --- | --- |
| **(Intercept)** | 0.661 | 0.056 | 758.6 | 11.790 | <0.001 |
| **age_5y** | 0.008 | 0.051 | 1040 | 0.147 | 0.883 |
| **age_6y** | -0.041 | 0.051 | 1040 | -0.798 | 0.425 |
| **age_7y** | -0.087 | 0.051 | 1040 | -1.719 | 0.086 |
| **trait_nice** | 0.179 | 0.045 | 554.0 | 3.963 | 0.001 |
| **gender_boy** | -0.015 | 0.052 | 1040 | -0.222 | 0.824 |
| **task order** | -0.028 | 0.021 | 552.0 | -1.343 | 0.180 |
| **parent_mother** | -0.014 | 0.033 | 552.0 | -0.438 | 0.661 |
| **age_5y:trait_nice** | -0.054 | 0.063 | 554.0 | -0.862 | 0.389 |
| **age_6y:trait_nice** | -0.028 | 0.063 | 554.0 | -0.454 | 0.650 |
| **age_7y:trait_nice** | 0.044 | 0.062 | 554.0 | 0.711 | 0.477 |
| **age_5y:gender_boy** | 0.049 | 0.074 | 1038 | 0.666 | 0.505 |
| **age_6y:gender_boy** | 0.126 | 0.072 | 1039 | 1.740 | 0.082 |
| **age_7y:gender_boy** | 0.156 | 0.072 | 1040 | 2.168 | 0.030 |
| **trait_nice:gender_boy** | -0.313 | 0.063 | 554.0 | -4.935 | <0.001 |
| **age_5y:trait_nice:gender_boy** | -0.022 | 0.090 | 554.0 | -0.239 | 0.812 |
| **age_6y:trait_nice:gender_boy** | -0.070 | 0.088 | 554.0 | -0.790 | 0.430 |
| **age_7y:trait_nice:gender_boy** | -0.150 | 0.088 | 554.0 | -1.750 | 0.089 |
